# Intestinal toxicity of the type B trichothecene mycotoxin fusarenon-X: whole transcriptome profiling reveals new signaling pathways

**DOI:** 10.1038/s41598-017-07155-2

**Published:** 2017-08-08

**Authors:** Imourana Alassane-Kpembi, Juliana Rubira Gerez, Anne-Marie Cossalter, Manon Neves, Joëlle Laffitte, Claire Naylies, Yannick Lippi, Martine Kolf-Clauw, Ana Paula L. Bracarense, Philippe Pinton, Isabelle P. Oswald

**Affiliations:** 1 Toxalim, Research Center in Food Toxicology, Université de Toulouse, INRA, ENVT, INP- PURPAN, UPS, F-31027 Toulouse, France; 2 Hôpital d’Instruction des Armées, Camp Guézo 01BP517 Cotonou, Benin; 3 Laboratory of Animal Pathology, Department of Veterinary Preventive Medicine, Universidade Estadual de Londrina, Londrina, Paraná Brazil; 4 Present Address: Université de Toulouse, Ecole Nationale Vétérinaire (ENVT), Toulouse, France

## Abstract

The few data available on fusarenon-X (FX) do not support the derivation of health-based guidance values, although preliminary results suggest higher toxicity than other regulated trichothecenes. Using histo-morphological analysis and whole transcriptome profiling, this study was designed to obtain a global view of the intestinal alterations induced by FX. Deoxynivalenol (DON) served as a benchmark. FX induced more severe histological alterations than DON. Inflammation was the hallmark of the molecular toxicity of both mycotoxins. The benchmark doses for the up-regulation of key inflammatory genes by FX were 4- to 45-fold higher than the previously reported values for DON. The transcriptome analysis revealed that both mycotoxins down-regulated the peroxisome proliferator-activated receptor (PPAR) and liver X receptor - retinoid X receptor (LXR-RXR) signaling pathways that control lipid metabolism. Interestingly, several pathways, including VDR/RXR activation, ephrin receptor signaling, and GNRH signaling, were specific to FX and thus discriminated the transcriptomic fingerprints of the two mycotoxins. These results demonstrate that FX induces more potent intestinal inflammation than DON. Moreover, although the mechanisms of toxicity of both mycotoxins are similar in many ways, this study emphasize specific pathways targeted by each mycotoxin, highlighting the need for specific mechanism-based risk assessments of *Fusarium* mycotoxins.

## Introduction

**Figure 1:**
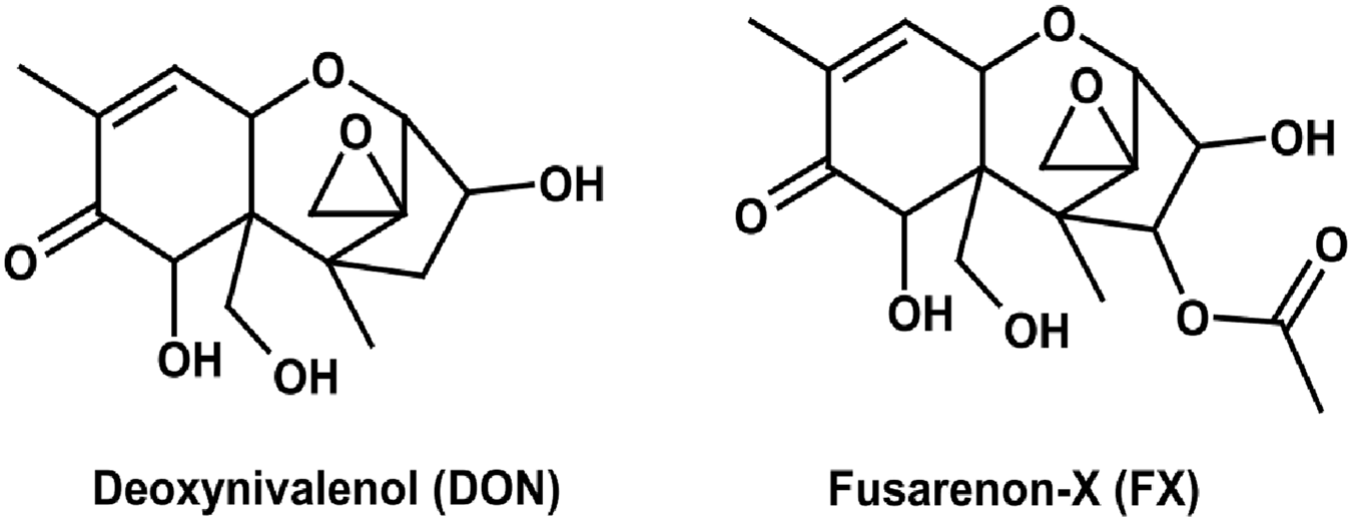
Chemical structures of DON and FX.

Trichothecene mycotoxins are of increasing concern with respect to food safety issues due to their frequent and global occurrence^1^. To date, only nivalenol and deoxynivalenol (DON), which are the most frequently occurring type B trichothecenes, are regulated^2^. Recent data have revealed that European consumers are also exposed to fusarenon-X (FX; 4-acetylnivalenol)^3–5^ (Fig. 1). Contamination of rice paddies in Japan has been reported, and up to 39% of Korean cereal-based products have tested positive for FX, which indicates that the occurrence of this mycotoxin in Asia and its significance for public health are probably underestimated^6, 7^.

Little is known about the effects of FX, and the available toxicity data are too limited to support derivation of a tolerable daily intake^8^. Exposure to FX is associated with diarrhea and extensive intestinal hemorrhaging with cellular destruction in the intestinal mucosa^9^. FX-induced diarrhea has been related to an increased cyclic AMP content of the intestinal mucosa^10^. The few available studies suggest a higher acute toxicity of FX compared with other trichothecenes^11^.

Because structurally related contaminants may co-occur, a toxic equivalency factor (TEF) can be defined to assess the combined risk^12^. This approach has been recently implemented for the *Fusarium* mycotoxin zearalenone and its modified forms^13^ as well as for the type A trichothecenes T2 and HT2 and their modified forms^14^. This approach has also been proposed for the type B trichothecene mycotoxins^15–17^. However, applying TEFs to trichothecenes would assume *inter alia* that these mycotoxins, including FX, have similar modes of action.

The intestinal mucosa is the first barrier against ingested chemical contaminants, including mycotoxins. Following ingestion of contaminated food, the intestine is exposed to high concentrations of mycotoxins^18, 19^. Using a porcine jejunal explant model, our research group reported the ability of DON and some trichothecenes to induce severe alterations of the intestinal morphology and an inflammatory response, which could result in increased susceptibility to intestinal inflammatory diseases^20–22^.

By giving a genome-wide view of biological responses, the transcriptional profiling may provide insight in pharmacological or toxicological mechanisms^23, 24^. The aim of this work was to analyze the intestinal toxicity of FX by comparison to DON in order to establish whether both toxins have similar modes of action or if their toxicities are related to different mechanisms. By combining a histo-pathological analysis, whole transcriptome profiling and qPCR analysis of selected genes, the present study was designed to obtain a global view of the toxicological alterations induced by FX in the intestine. In addition to the overlapping biological effects of FX and DON, we report for the first time several signaling pathways that discriminate the transcriptomic fingerprints of the two mycotoxins.

## Results and Discussion

### Intestinal exposure to FX induces more severe histological alterations than exposure to DON

A histo-morphometric analysis was conducted on pig intestinal explants exposed to FX and its well-characterized congener DON to obtain a comparative appraisal of the tissue alterations induced by both trichothecene mycotoxins.

**Figure 2:**
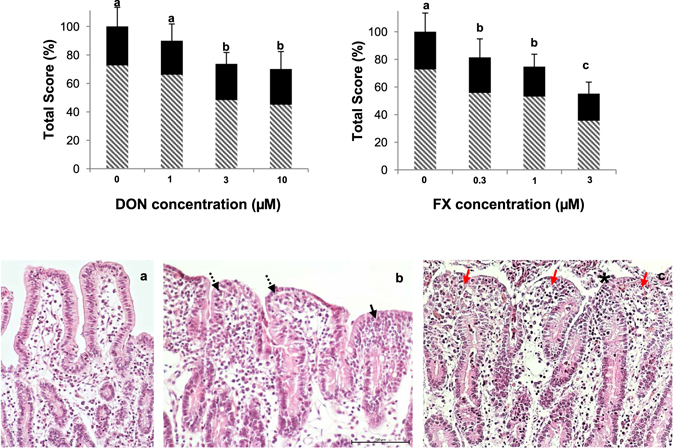
Effect of exposure of the jejunal explants to different levels of DON or FX. Top panel: Morphological scores (black) and lesional scores (striped) observed following the 4-hour exposure to DON or FX. The values are the mean percentage ± SD. ^a,b^Mean values with different letters were significantly different (p < 0.05). Tukey test. Bottom panel: Histological aspects. (**a**) Control explant. Villi lined with columnar enterocytes (HE, 20x); (**b**) 3 µM DON-exposed explant. Moderate fusion (black arrow) and villi showing cubic epithelial cells (dotted arrow) (HE, 20x); (**c**) 3 µM FX-exposed explant. Villi showing severe apical necrosis (red arrow) and diffuse cellular debris (*) (HE, 20x).

The 4-hour exposure to 3 or 10 µM of DON resulted in a significant histo-morphological alteration of the explants (Fig. 2I). The main morphological lesions were the coalescence of villi with moderate fusion. Intestinal alterations also included cubic epithelial cells, edema in the *lamina propria*, villi atrophy and apical denudation of villi with a focal loss of apical enterocytes (Fig. 2IIb). In line with previous data, exposure of intestinal explant to 10 µM DON, induces histological lesions without altering integrity of the tissue^25, 26^.

Similar to DON, significant decreases in the histo-morphological score were observed in the explants treated with FX. The scores were reduced by 18, 25 and 45% in explants treated with 0.3, 1 and 3 µM of FX, respectively, compared with the approximately 30% reduction in the histo-morphological score induced by 10 µM of DON (Fig. 2I). The explants exposed to FX displayed lesions similar to the DON-treated explants. However, villi with an absence of epithelia, severe atrophy, diffuse cellular debris along the surface of the intestinal mucosa and a reduction in the villi numbers were observed at the highest FX dose (Fig. 2IIc). The low tested doses of FX (0.3 to 3 µM) already induced severe lesions on intestinal mucosa, suggesting that higher doses may induce a total loss of tissue integrity and would interfere with histo-morphological score.

Considering both the relevance of the pig model for humans and the high sensitivity of pigs to mycotoxins^27, 28^, pig intestinal tissue culture represents a useful tool for investigations of the intestinal toxicity of these food contaminants. Using a pig jejunal tissue culture model, our lab previously demonstrated the ability of DON and its acetylated derivatives to induce severe alterations of the intestinal morphology^21, 26^. In the present study, we demonstrated that although FX induced a type of intestinal histological alteration that was similar to the alterations observed with DON, the lesions were more pronounced with FX.

The explant model oversimplifies the gastro-intestinal environment, especially regarding the absence of microbiota, and as a consequence it could not take into account possible interactions between mycotoxins and microbiota. Few publications investigated such interactions in the gastro-intestinal environment^29^. Nevertheless, the transformation of trichothecenes, by the intestinal microbiota of monogastric species is known to occur in a very limited extent^30^; and especially in piglets, the high oral bioavailability of FX suggests that this mycotoxin largely escapes the gastrointestinal transformation^31^. Furthermore, the monitoring of the kinetics of depletion of FX in different tissues pointed that substantial amounts of FX were still found in the intestinal tissue 24 hours after a single oral administration, which indicates that the intestine may have to face the deleterious effects of the dietary exposure to FX^31^.

A low mean contamination level of 27.2 µg FX/kg, which corresponded to daily intakes ranging from 0.004–0.013 µg/kg bw/day to 0.009–0.032 µg/kg bw/day for adults and children, respectively, was reported in Spanish breakfast cereals^5^. However, a 100 times higher FX contamination level was observed in lodged and water-damaged domestic rice in Asia^7^. Assuming that a human adult weighting 70 kg has a small intestinal volume of 1 L, the 0.3–10 µM FX concentration range tested in this study is realistic in the human gut^32^. The main findings concerning the toxicological effects of FX to date are summarized in Supplementary Table S1. These effects encompass immunosuppression as well as reproduction and developmental toxicity. Forty-eight hours following oral administration, FX could still be readily detected in the plasma samples of piglets, even though it was partially converted into NIV (Saengtienchai *et al*., 2014). Likewise, monitoring the kinetics of depletion of this mycotoxin in different tissues in the same study indicated that substantial amounts of FX were still found in the intestinal tissues 24 hours after oral administration. This relatively long-term exposure following a single oral administration suggests that the intestine may be hit by dietary as well as systemic FX. In line with the *ex vivo* observations presented here, a marked shortening of the intestinal villi in the jejunum and the ileum has been reported 24 hours following a single intra-peritoneal injection of 1 mg/kg of FX to rats^33^. FX also induced extensive hemorrhaging in the intestine with cellular destruction and karyorrhexis of the intestinal mucosa in mice, and apoptotic cell death in the rat gastric mucosa, in acute and sub-acute toxicity studies^9, 34, 35^. Our results emphasize that the intestine is a front-line target organ after dietary exposure to FX.

The toxicity rankings for FX and DON based on the histo-morphometric analysis indicate greater intestinal toxicity of FX, which is in accordance with the few available comparative toxicity studies of these toxins, including cytotoxicity toward intestinal cell lines^36, 37^, emesis^16, 17^, and anorexia^38^.

### Gene expression profiles of intestinal explants exposed to FX or DON: intestinal inflammation is the hallmark of the molecular toxicity of both toxins

To obtain an in-depth view of the intestinal toxicity of FX, we evaluated the global impact of FX (2 µM and 10 µM) compared with DON (10 µM) on the intestinal transcriptome using a porcine pan-genomic microarray^28^. The 2-µM FX concentration was included in order to investigate the transcriptomic impact of FX and DON at concentrations inducing comparable histological tissue damages (Fig. 2).

**Figure 3:**
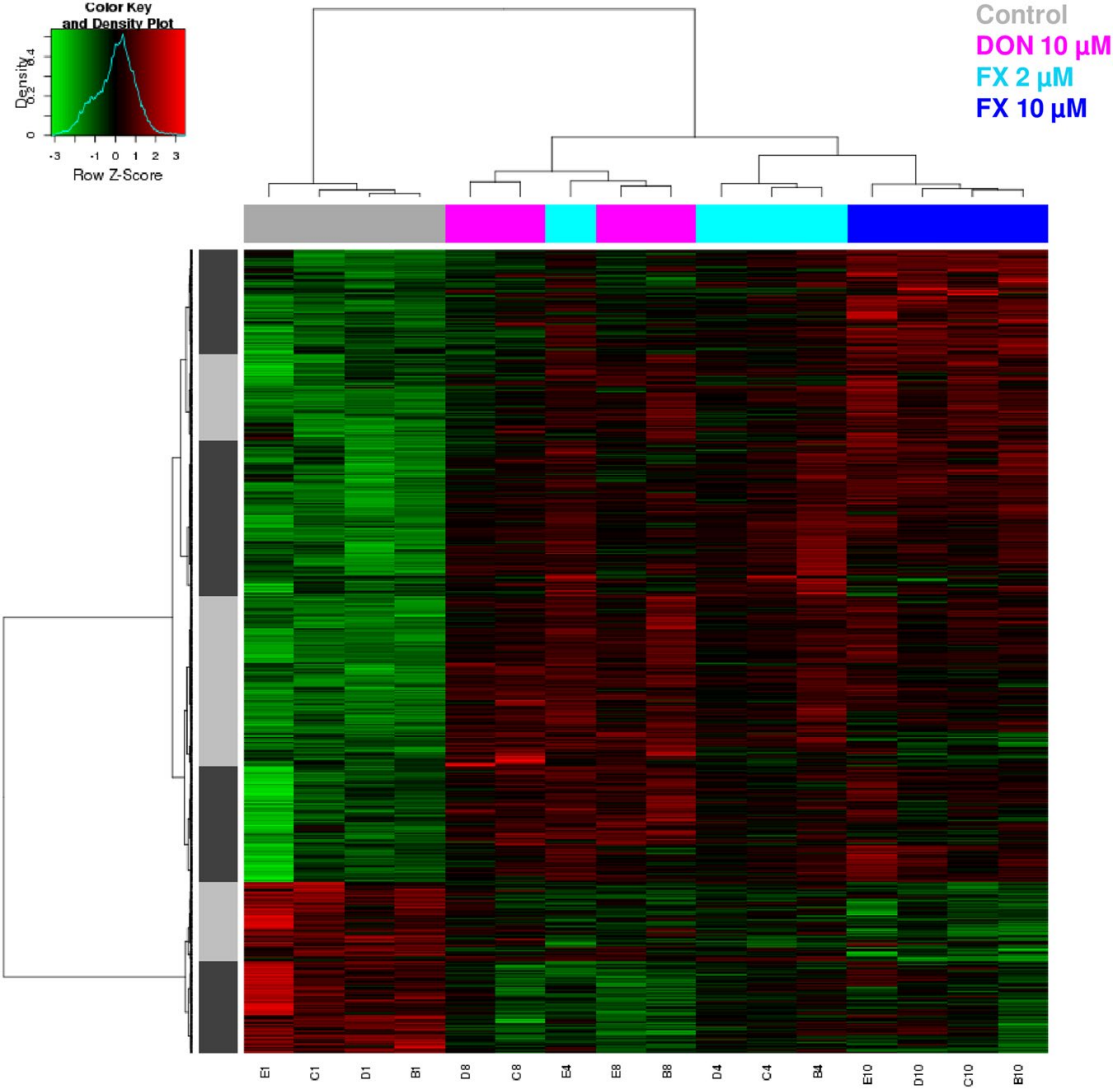
Gene expression profiles of intestinal explants exposed to DON or FX. Heatmap representing differentially expressed probes between the control, DON, and FX conditions. Jejunal explants from 4 different animals were exposed for 4 hours to DMSO (vehicle), 10 μM DON (as a benchmark for the toxicity of TCTBs), or 2 or 10 µM FX. Gene expression was analyzed with a 60 K microarray. Red and green colors indicate values above and below the mean (average Z-score), respectively. Black indicates values close to the mean.

Exposure to FX and DON resulted in clear modification of the transcriptomic profiles of the intestine (Fig. 3). A total of 738 genes were differentially expressed (DE) (adjusted P value < 0.05), with fold-change values ranging from −2.17 to 9.8. The number of DE genes increased from 372 to 517 following treatment with 2 µM and 10 µM of FX, whereas 411 genes were differentially expressed in response to 10 µM of DON. The regulated genes upon exposure to 2 µM FX, 10 µM FX and 10 µM DON are presented in Supplementary Table S2. These genes encode cytokines, transporters, transcription regulators, G-protein coupled receptors, transmembrane receptors, ligand-dependent nuclear receptors, growth factors and enzymes.

**Figure 4:**
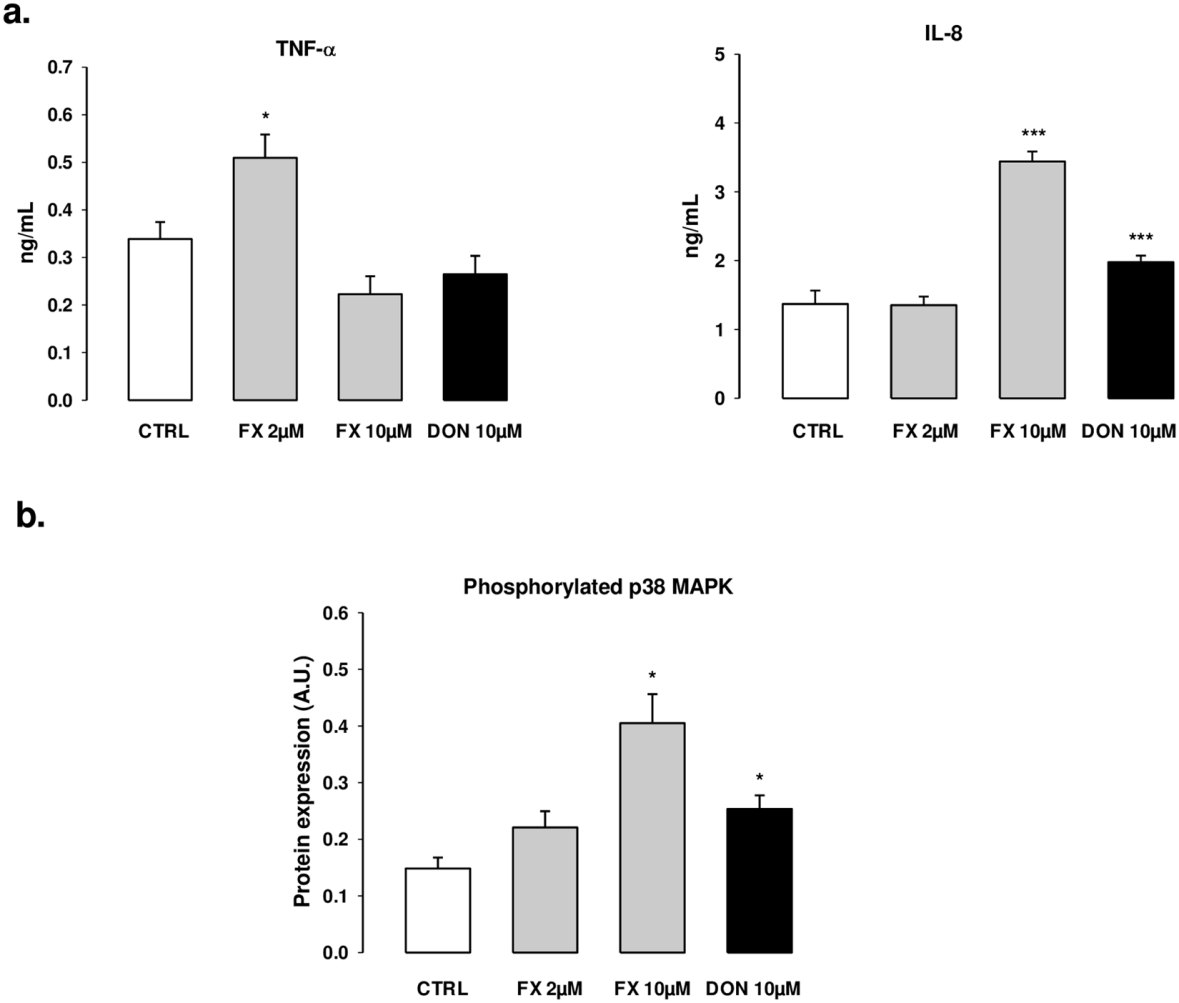
Intestinal inflammation is the hallmark of the molecular toxicity of DON and FX. IPEC-J2 cells differentiated on inserts were cultured in presence of 2 or 10 µM of FX, or 10 µM of DON, or DMSO for 24 h or 48 h. (Panel a) TNF and IL-8 production in the cell culture supernatants were quantified by ELISA. (Panel b) Cell lysates were analyzed by western blot for expression of phosphorylated p38 MAPK, using β-actin as a protein loading control. The normalized expression data are presented. The results for ELISA and western blot are expressed as mean ± SEM of 3 independent experiments, *p < 0.05, **p < 0.01, ***p < 0.001.

For both FX and DON, the top up-regulated genes in the intestinal explants were mainly related to inflammation. The interleukin 1 alpha and beta (*IL-1α* and *IL-1β*), chemokine ligand 20 (*CCL20*), and tumor necrosis factor alpha-induced protein 3 (*TNFAIP3*) genes were the most up-regulated genes. Other inflammatory genes in the top up-regulated category included the chemokines *CXCL2* and *IL8*, the cytokines *TNF*, *CSF2*, *IL-17A* and *IL-22*, and NF-κB inhibitor alpha (*NF-κBIA*). The *AREG* gene, which can be expressed by multiple populations of activated immune cells under inflammatory conditions, was also up-regulated. One notable finding is the over-expression of the Kruppel-like transcription factor 10 (*KLF10*), which functions both as an inhibitor of cell proliferation and an inducer of apoptosis, resulting in enhanced immune system and inflammatory processes. Using the differentiated porcine non-transformed intestinal cell line, IPEC-J2, we further verified that modifications of gene expression were translated at the protein level. The quantification of TNF-α and IL-8 in cell supernatants showed a 2-fold induction following the FX exposure (Fig. 4 Panel a), versus 3–4 fold induction at the gene level (Supplemental Table S2) and confirmed previous data obtained on DON^20, 26^.

**Figure 5:**
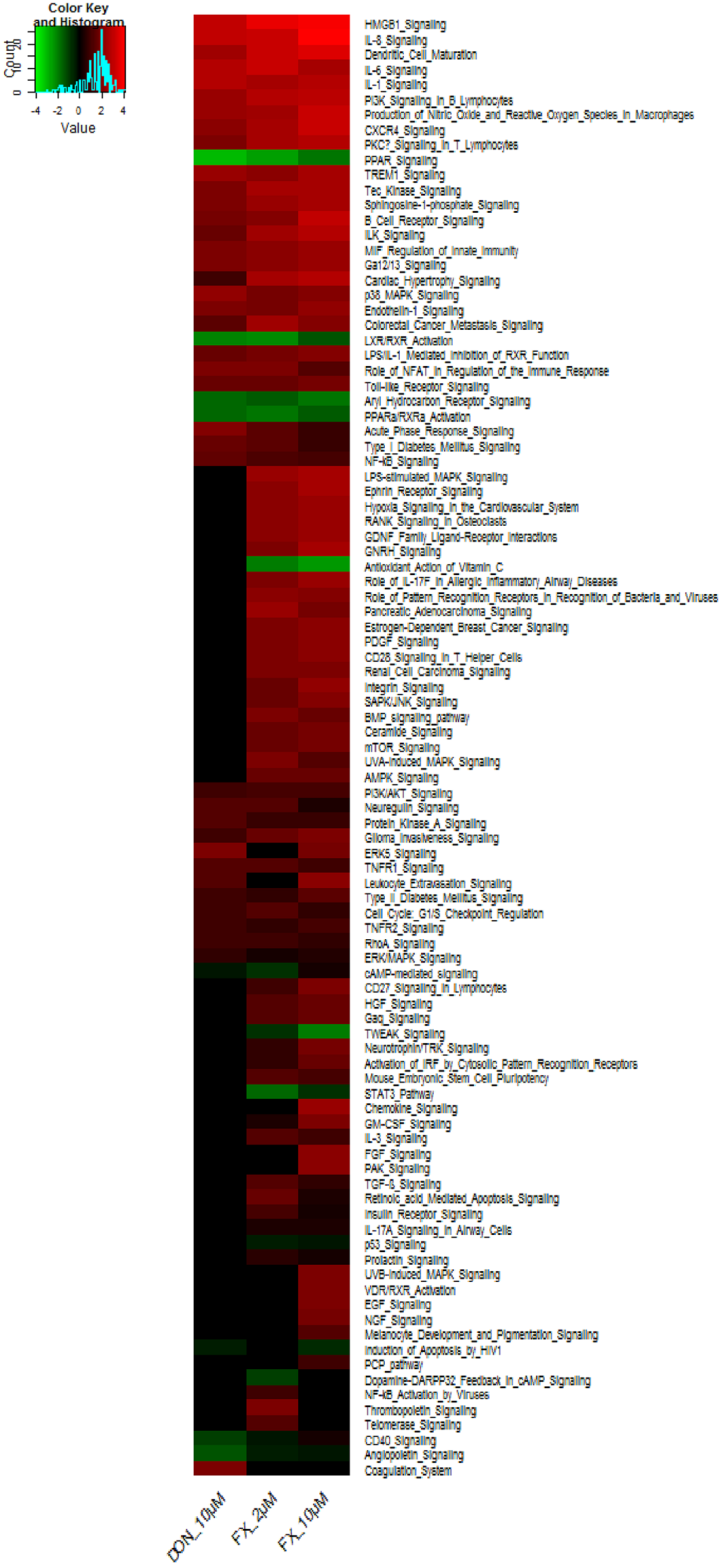
Heatmap representation of the intestinal modulation of signaling pathways by the trichothecene mycotoxins FX and DON. Only the signaling pathways with an IPA Z-score with an absolute value ≥ 2 were preserved.

Using the Ingenuity Pathway Analysis (IPA) software, a heatmap was constructed from a signaling pathway-based functional analysis of the DE genes upon intestinal exposure to FX and DON (Fig. 5). This analysis confirmed that both FX and DON primarily activated immunity/inflammation-related canonical signaling pathways, including the IL-6, IL-1, IL-8, HMGB1, and Toll-like receptor pathways.

In addition to inflammatory genes, other up-regulated genes upon intestinal exposure to FX and DON included the dual specificity phosphatase family member 6 (*DUSP6*, also named MAP kinase phosphatase 3 (*MKP3*)), the DNA damage gene markers *GADD45A* and *GADD45G* and the cytochrome P450 family 1 member A1-encoding gene *CYP1A1*.

The top down-regulated genes for both trichothecenes included *ABCC2*, which encodes the ATP binding cassette superfamily subfamily C member 2 efflux protein, *SLC1A1*, *SLC9A2* and *SLC15A1*, which encode proteins involved in the transport of various molecules across the membranes, and the *MGAM* gene, which encodes the brush border membrane enzyme maltase-glucoamylase.

FX has been previously reported to induce abdominal and systemic inflammation in mice^39, 40^. The results presented in this study shed light on the signaling pathways involved in the inflammatory response following intestinal exposure to FX. IL-6 is generated upon activation of the pattern recognition receptors (PRRs) by pathogen-associated molecular patterns or damage-associated molecular patterns (DAMPs) and functions as a mediator that issues a warning signal in case of infectious lesions or tissue damage^41^. In mononuclear cell cultures, HMGB1 has been shown to induce the release of nitric oxide and the secretion of TNF-α, which are key mediators in gut inflammation^42, 43^. HMGB1 belongs to a family of endogenous compounds termed alarmins that can be released into the extracellular milieu during states of cellular stress or injury that lead to infectious or non-infectious conditions^44^. The ribosomal RNA cleavage previously reported during DON-induced ribotoxic stress could also apply to FX and play a role in the cellular injury leading to DAMP-mediated PRR activation of IL-6 and HMGB1 signaling^45^. Interestingly, and in line with this hypothetical cytosolic sensing of cleaved RNA, the aryl hydrocarbon receptor (AhR) signaling pathway was also down-regulated by FX under our experimental conditions (Fig. 4). Yamada *et al*.^46^ recently established that endogenous AhR signaling regulated the activation of type I interferon production mediated by the cytosolic RNA sensor RIG-1.

Other inflammation/immunity-related pathways stimulated by FX include the sphingosine-1-phosphatase (S_1_P), p38 MAPK, CXCR4, and NF-κB signaling pathways (Fig. 5). The roles of S_1_P signaling as a significant modulator of a number of inflammation-related functions have recently emerged^47^. These functions include cytokine and adhesion molecule expression, lymphocyte maturation, differentiation and trafficking, and mast cell trafficking. The production of pro-inflammatory cytokines under pathological conditions, including inflammatory bowel disease, is postulated to be regulated in part by the p38 MAPK pathway^48^. In the present study, we observed that FX activates the phosphorylation of p38 MAPK (Fig. 4 Panel b) as previously demonstrated for DON^26^.

The nuclear factor-κB (NF-κB) signaling pathway regulates immune responses and is implicated in the pathogenesis of many inflammatory diseases^49, 50^. The CXCR4 signaling plays a prominent function in orchestrating both innate and adaptive immune responses through the following mechanisms: (i) by regulating leucocyte trafficking and distribution to and from peripheral tissues, (ii) by participating in lymph node organization, and finally (iii) by sustaining T cell priming^51^.

### FX and DON down-regulate the PPAR and LXR-RXR signaling pathways that control lipid metabolism

The comparative functional analysis of DE genes in the intestine upon exposure to FX and DON also revealed repression of several pathways that control lipid metabolism, such as the peroxisome proliferator-activated receptor (PPAR)-, liver X receptor (LXR)- and retinoid X receptor (RXR)-dependent pathways (Fig. 5). The repression of these signaling pathways was associated to a 3-fold increase of *CYP1A1* gene expression (Supplemental Table S2), which supports the implication of nuclear receptors in the modulation of the P450 activity in response to specific stimuli^52^.

The PPAR/RXR and LXR/RXR heterodimers act as sensors of lipids that are derived from both the diet and intracellular metabolism and thereby regulate diverse aspects of cholesterol and fatty acid homeostasis^53^. To the best of our knowledge, this study is the first report to indicate that trichothecenes interfere with lipid homeostasis. Lipid metabolism is linked to immunity, and studies have demonstrated that pathogens, including *Escherichia coli*, *Chlamydia pneumonia* and influenza A virus, can down-regulate LXR signaling^54^. LXRs antagonize the uptake of modified lipids, such as oxidized LDL, by macrophages in the vascular wall, which constitutes the first event in the formation of foam cells and the subsequent onset of atherosclerotic lesions^55^. The implications of down-regulation of the lipid homeostasis signaling pathways by trichothecenes warrants further investigation.

### Specific pathways discriminate the intestinal transcriptomic fingerprints of FX and DON

**Figure 6:**
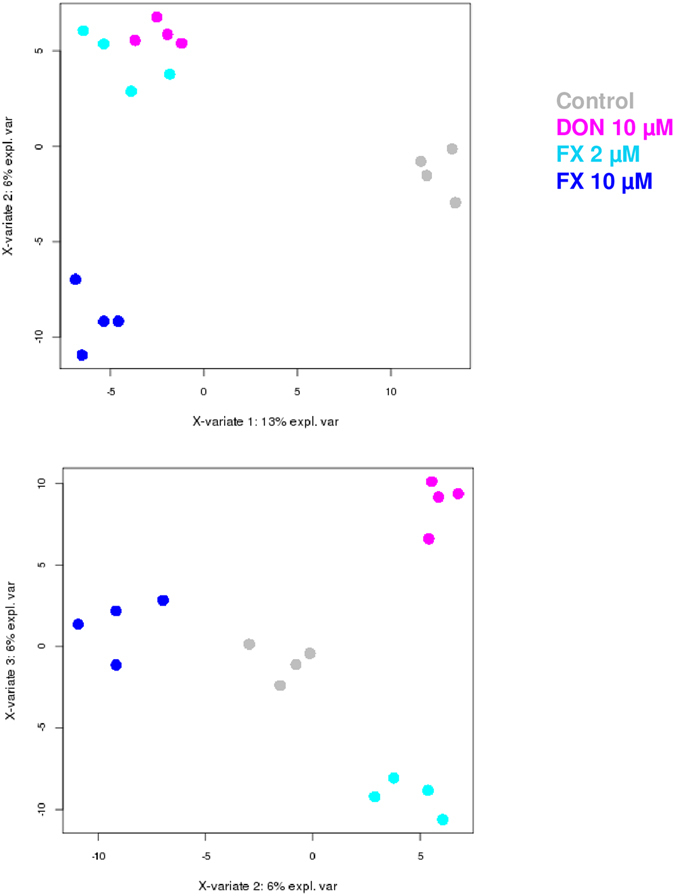
Scatter plots of the jejunal explant samples in the control, DON 10 µM, FX 2 µM and FX 10 µM conditions using the first three components from the sPLS-DA analysis. Scatter plots were constructed using the first three hidden components selected by the sPLS-DA. These hidden components are obtained as linear combinations of the microarray probes using the estimated regression weights.

**Figure 7:**
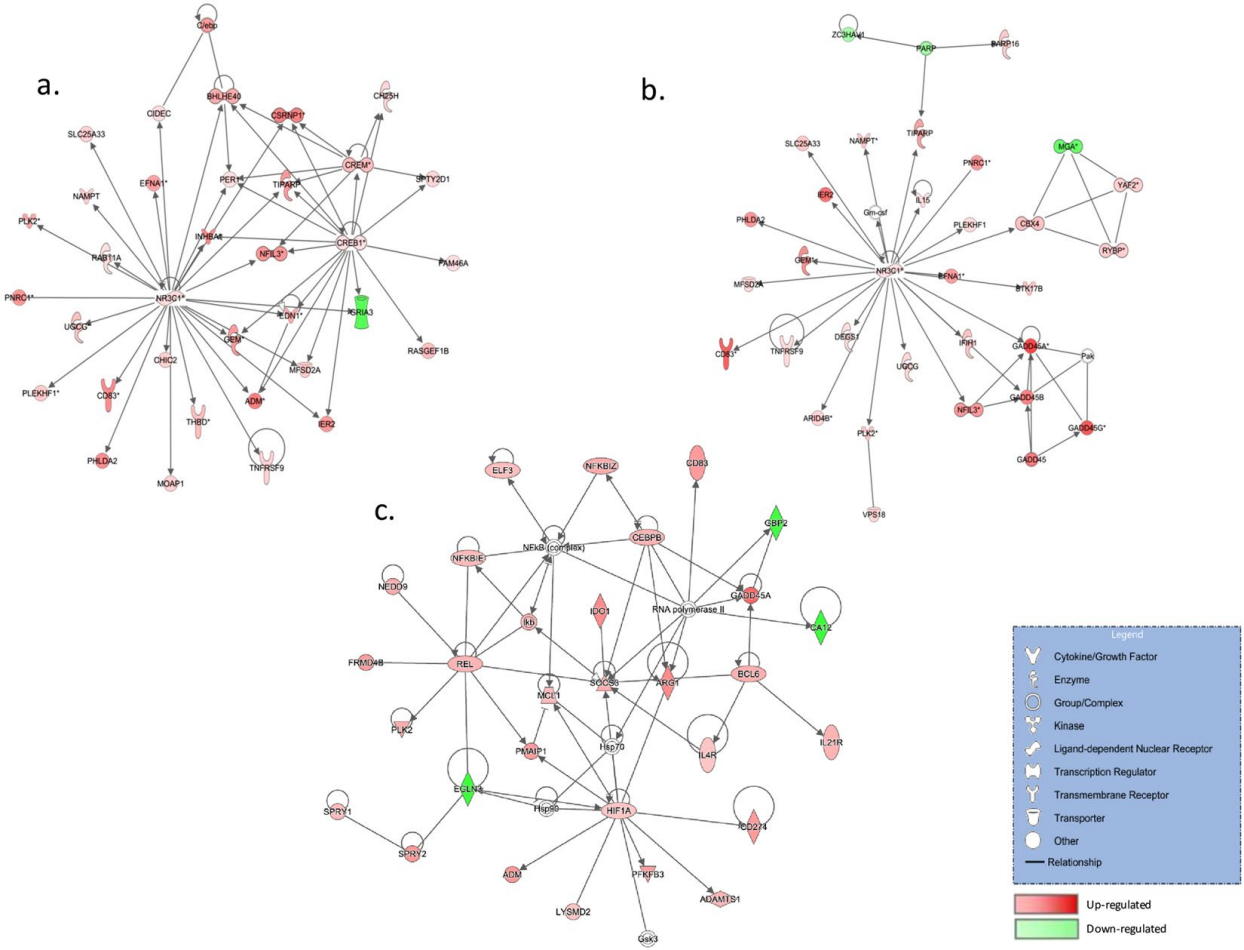
Network analysis for the differentially expressed genes (DEGs) in the intestine following exposure to FX or DON. To create gene networks, DEGs following exposure to FX 2 µM (**a**), FX 10 µM (**b**) or DON 10 µM (**c**) were overlaid onto a global molecular network that was developed from information contained in the Ingenuity Knowledge Base. The IPA Network Generation Algorithm links genes based on experimentally observed interactions and orders these genes based on their interconnectedness. Generally, the more interactions with other network members, the more central a gene will be in a network. In the networks, the edges or arrows indicate gene interactions. Only direct interactions have been considered. Red nodes represent up-regulated genes, and green nodes represent down-regulated genes identified in our differential expression analysis. Darker node colors indicate more extreme (high or low) up- or down-regulation of the respective gene. White nodes represent genes that were not originally identified in our microarray analysis but were are predicted by IPA to be involved in the network.

In addition to their shared features, this study unveiled specific traits associated with the toxicity of FX. Figure 6 presents a Sparse Partial Least Squares Discriminant Analysis (sPLS-DA) of the intestinal transcriptomic profiles under the 2 µM FX, 10 µM FX and 10 µM DON conditions. Dimension 1 of the sPLS-DA discriminates the control from the mycotoxin-treated conditions, whereas Dimension 2 discriminates 10 µM of FX from 2 µM of FX and 10 µM of DON and Dimension 3 emphasizes differences in gene expression between the 2 µM FX and the DON conditions. Figure 6 also illustrates that histological damages are correlated with transcriptomic results, since the FX 10 µM condition was discriminated from other mycotoxin treatments. Nonetheless, Dimension 3 of sPLS-DA clearly discriminates FX 2 µM and DON 10 µM that have similar histological impacts, and indicates that the transcriptomic fingerprint is also linked to the toxin. These distinctive transcriptomic profiles are also highlighted by the IPA gene network-based functional analysis of the DE genes (Fig. 7). The top network generated from the DE gene list for 2 µM FX exposure was centered on three transcription factors: *NR3C1*, which is involved in the inflammatory response, *CREB1*, which encodes a protein that induces the transcription of genes in response to hormonal stimulation of the cAMP pathway, and *CREM*, which also encodes an important component of cAMP-mediated signal transduction (Fig. 7 Panel a). As expected, the top network generated from the DE gene list for 10 µM of FX was also centered on *NR3C1* (Fig. 7 Panel b). Following DON exposure, in addition to the suppressor of cytokine signaling *SOCS3* and NF-κB subunit *REL*, which are associated with inflammation, the top network was centered on the hypoxia-inducible transcription factor *HIF1A*, which is associated with energy metabolism, angiogenesis, and apoptosis (Fig. 7 Panel c). Unsurprisingly, the cAMP-mediated signal transduction associated with *CREB1* and *CREM* appeared to be pivotal in the network analysis for 2 µM of FX, because increased cAMP levels have been reported in the rat intestinal mucosa upon exposure to FX^10^. The important role of cAMP in the pathophysiology of FX, in contrast to DON, could partly explain why Dimension 2 of the sPLS-DA suggested high similarity of the transcriptomic profiles of 2 µM of FX and 10 µM of DON, whereas Dimension 3 discriminated both conditions.

Finally, the most clear indications of the differences between DON and FX are given by the signaling pathways based on the functional analysis of the DE genes and its heatmap representation (Fig. 5). Several signaling pathways, including the VDR/RXR activation, ephrin receptor, GNRH, integrin and ceramide signaling pathways, were specific for intestinal exposure to FX, whereas down-regulation of the angiopoietin and CD40 signaling pathways in the intestine was exclusively observed with DON.

The VDR/RXR activation under the FX condition may indicate that this mycotoxin has a more pleiotropic impact on the intestinal transcriptome than DON. The vitamin D receptor (VDR) can form a heterodimer with the 9-cis retinoid X receptor (RXR) to regulate the expression of vitamin D target genes^56^. In addition to controlling bone mineralization, the physiological impact of vitamin D is very broad, as attested by chromatin immunoprecipitation sequencing studies in different human cell culture models, which have identified more than 23,000 non-overlapping loci for the receptor^57^.

Our results highlight that FX and DON, which only differ structurally by O-acetylation on C4, diverge in their biological activity in the intestine. These findings are in line with the differences in gene expression patterns reported for DON and FX in a *Saccharomyces cerevisiae* strain toxicity test^58^.

Group health-based guidance values in combination with relative potency factors were recently applied to the *Fusarium* mycotoxin zearalenone and its structurally related compounds as well as the type A trichothecenes T2 and HT2 and their modified forms^13, 14^. Applying this strategy to the type B trichothecenes would assume *inter alia* that these mycotoxins, including DON and FX, have similar modes of action. Our data show that in addition to their overlapping biological effects, the toxicities of DON and FX significantly deviate from one another. This finding suggests that the toxicity evaluations of DON and FX should be conducted separately.

### Dose-response analysis of gene expression confirms the high intestinal toxicity of FX

As inflammation/immunity appeared to be the hallmark of the transcriptomic impact of FX, the pro-inflammatory potency of FX was further analyzed by qPCR.

**Figure 8:**
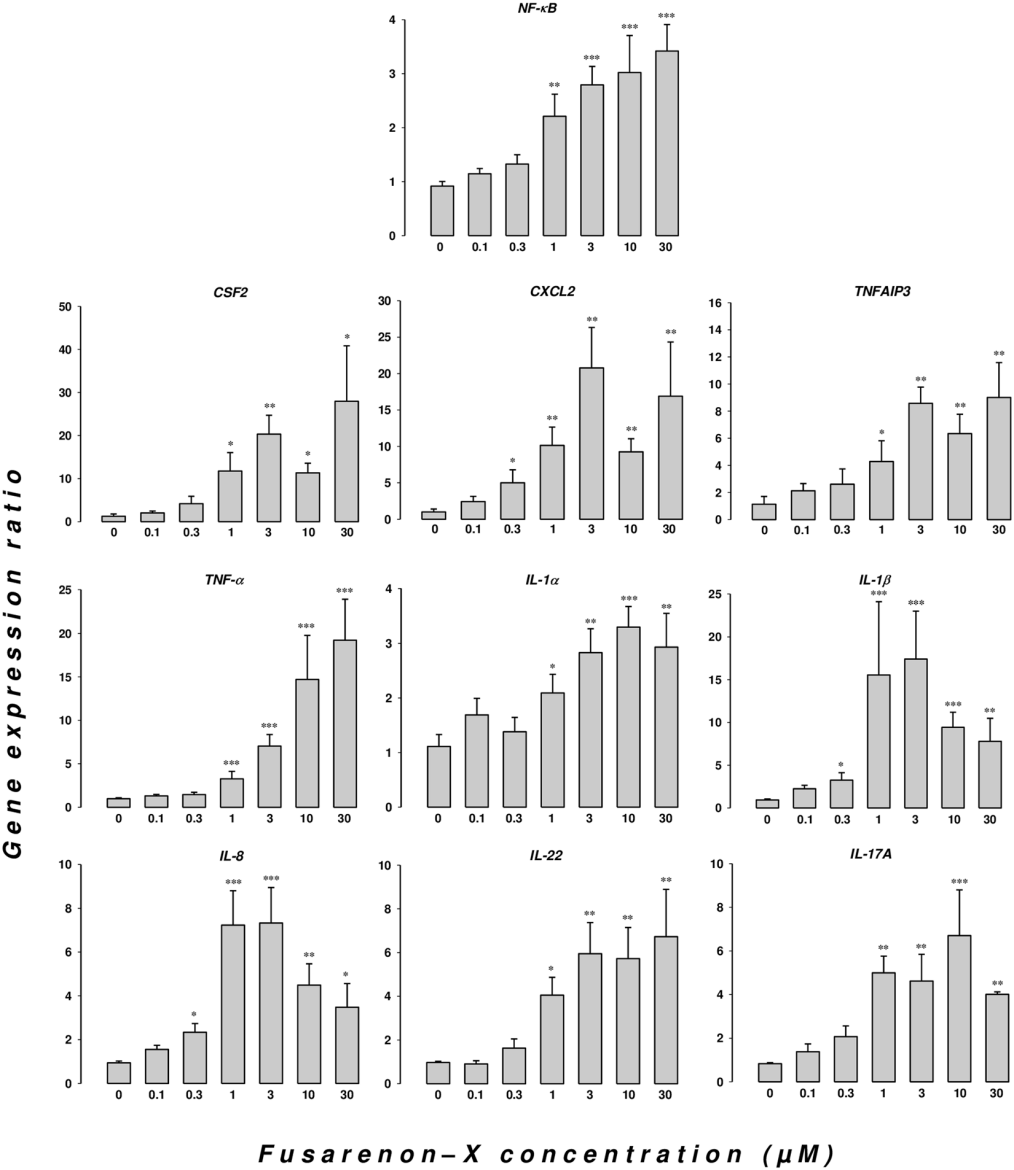
Dose-dependent regulation of inflammatory genes in the intestine upon exposure to FX. Dose-responses for the FX regulatory gene expression levels in the pig intestine. Jejunal explants from 6 piglets were exposed for 4 hours to graded levels of the trichothecene mycotoxin FX. The mRNA expression levels of *NF-κB*, *CSF2*, *CXCL2*, *TNFAIP3*, *TNF-α*, *IL-1α*, *IL-1β*, *IL-8*, *IL-17A* and *IL-22* were measured by quantitative RT-PCR and analyzed using the REST© 2009 software (Qiagen, Valencia, CA, USA). *β2-Microglobulin* and *RPL-32* were used as the internal references. The data are the mean ± SEM (n = 6 animals). The symbols ^*^, ^**^, and ^***^ indicate significant differences in gene expression ratios for the treated conditions compared to the control conditions (p < 0.05, p < 0.01, and p < 0.001, respectively).

The mRNA expression levels for the key NF-κB transcription factor and 9 cytokine and inflammation-related genes (*IL-1α*, *IL-1β*, *IL-8*, *TNF-α*, *CXCL2*, *CSF2*, *TNFAIP3*, *IL-17A* and *IL-22*) were measured on pig jejunum explants exposed for 4 hours to graded levels of FX. As shown in Fig. 8, FX induced a dose-dependent up-regulation of the mRNA expression levels of all tested genes. The genes encoding CSF2, CXCL2, TNFAIP3, TNF-α, IL-1β, IL-8, IL-17A and IL-22 showed higher levels of activation than the genes encoding the transcription factor NF-κB/p50 and the cytokine IL-1α.

A benchmark dose analysis was performed to derive the threshold doses for the up-regulation of pro-inflammatory genes by FX in the intestine. Selected dose-response curves and the fitting models for the mRNA expression data are presented in Fig. S1. The BMD and lower confidence bound (BMDL) values derived for the up-regulation of the expression of the transcription factor NF-κB/p50 gene and the inflammatory genes *IL-1β*, *CXCL2*, *CSF2*, *TNFAIP3*, *IL-8*, *TNF-α*, *IL-17A* and *IL-22* are presented in Table 1. A dose of FX as low as 0.04 µM constituted the threshold for activation of the pro-inflammatory *IL-1β* gene in the intestinal tissue. The BMDL values for *IL-1β*, *CXCL2*, *CSF2*, *TNFAIP3*, *IL-8* and *IL-17A* expression (0.04 to 0.08 µM) were substantially lower than the values obtained for *NF-κB/p50*, *TNF-α*, and *IL-22* (0.11 to 0.34 µM).

**Table 1:** Benchmark doses for the intestinal up-regulation of the *NF-κB* transcription factor and pro-inflammatory cytokine gene expression by FX.

Genes	*BMD* (*µM*)	*BMDL* (*µM*)
*NF-κB*	*0.73*	*0.34*
*TNF-α*	*0.57*	*0.34*
*TNFAIP3*	*0.3*	*0.07*
*CXCL-2*	*0.13*	*0.05*
*CSF2*	*0.18*	*0.07*
*IL-1β*	*0.14*	*0.04*
*IL-8*	*0.14*	*0.08*
*IL-17A*	*0.15*	*0.05*
*IL-22*	*0.5*	*0.33*

This study is the first report of dose-dependent expression of *NF-κB* mRNA transcripts upon exposure to a trichothecene mycotoxin. This finding corroborates the selective increase in binding activity of the NF-κB/p65 subunit observed for DON^59^ and provides evidence of transcriptional regulation of the NF-κB signaling pathway by trichothecenes in addition to the previously proposed mechanism based on inhibition of IκBα protein synthesis by DON^60^. These data also confirm the high toxicity of FX, with BMDL ranging from 0.04 µM to 0.34 µM depending on the cytokine considered. Indeed, we previously reported higher benchmark doses for the intestinal up-regulation of pro-inflammatory gene expression by DON with BMDL values for intestinal inflammation ranging from 0.16 µM to 1.86 µM^61^. FX also appeared much more toxic than its biosynthesis derivative NIV (intestinal pro-inflammatory BMDL values ranging from 0.73 to 4.11 µM)^61^. The BMD determination for the induction of anorexia in the mink also indicated a higher toxicity of FX than DON^15^. Altogether, these data confirm that intestinal inflammation is a very sensitive endpoint for the health risk assessment of the trichothecenes; the effective doses reported for this group of mycotoxins on critical functions of the intestine have been estimated to be greater than 1 µM until recently^62^.

## Conclusion

The present study investigated the intestinal toxicity of FX compared with DON. Exposure to FX resulted in more severe histo-morphological alterations than exposure to DON. Transcriptomic profiling revealed that the early alterations in the responses to both mycotoxins were primarily linked to a pro-inflammatory effect. The FX doses associated with the deleterious pro-inflammatory effects were clearly below the known effective doses for the toxicity of other type B trichothecenes targeting other critical functions. We also report that in addition to their overlapping inflammatory effects, the toxicity of DON and FX significantly deviate from one another, especially regarding the CD40, ephrin receptor, GNRH, integrin and ceramide signaling pathways and VDR/RXR activation.

## Material and Methods

### Toxins

Deoxynivalenol (DON) was purchased from Sigma (St. Quentin Fallavier, France), and fusarenon-X (FX) was purchased from Wako Pure Chemical Industries Ltd. (Osaka, Japan). The toxins were dissolved to 60 mM in dimethylsulfoxide (DMSO) and stored at −20 °C before dilution in complete culture media.

### Intestinal Cell Line

IPEC J2 cells, derived from the small intestine of a newborn unsuckled piglet were grown and differentiated as already described^26^. Cells were treated with toxins (2 or 10 µM FX, 10 µM DON) or vehicle for 24 h or 48 h before supernatants were collected for cytokine analysis, and cells harvested for MAPK immunoblotting. Three independent experiments were performed for each cell culture condition.

### Jejunum explant culture

Jejunal explants were obtained from 5-week-old crossbred castrated male piglets (n = 6) as previously described^21, 28^. The experiment was conducted under the guidelines of the French Ministry of Agriculture for animal research. All animal experimentation procedures were approved by the Ethics Committee of Pharmacology-Toxicology of Toulouse-Midi-Pyrénées in animal experimentation (Toxcométhique) (N°: TOXCOM/0017/IO PP) in accordance with the European Directive on the protection of animals used for scientific purposes (Directive 2010/63/EU). Three authors (I.A.K, I.P.O. and P.P) have an official agreement with the French Veterinary Services allowing animal experimentation. The explants were treated for 4 hours at 39 °C with 10 μM of DON or graded concentrations of FX (0.1–10 µM) or vehicle (DMSO) in complete medium. After incubation, the treated explants were fixed in 10% formalin (Sigma-Aldrich) for the histological analysis or stored at −80 °C for RNA extraction.

### Histo-morphometric analysis

Explants fixed with 10% formalin for 24 hours were dehydrated and embedded in paraffin wax (Labonord, Templemars, France) according to standard histological procedures. Sections (5-μm-thick) were stained with hematoxylin and eosin (Sigma-Aldrich) for histo-pathological assessment. The histological findings were scored based on histological changes and the severity of the lesions as previously described^21, 28^.

### RNA extraction

Jejunal explants were lysed in 1 mL of Extract All reagent (Eurobio, Les Ulis, France) with ceramic beads (Bertin Technologies, St. Quentin en Yvelines, France). Total RNA was extracted according to the manufacturer’s recommendations as previously described^63, 64^. The RNA concentration was determined by measuring the optical density at 260 nm (OD260), and the RNA integrity was assessed using both NanoDrop spectrophotometric analysis (Nanodrop ND1000, Labtech International, Paris, France) and Agilent capillary electrophoresis (Agilent 2100 Bioanalyzer, Agilent Technologies Inc., Santa Clara, CA, USA). The mean ( ± SD) RNA Integrity Number (RIN) of these mRNA preparations was 6.85 ± 0.8.

### Microarray processing and functional analysis of expressed genes

The microarray GPL16524 (Agilent technology, 8 × 60 K) used in this experiment consisted of 43,603 spots derived from the 44 K (V2:026440 design) Agilent porcine-specific microarray. This microarray was enhanced with 9,532 genes from adipose tissue, 3,776 genes from the immune system and 3,768 genes from skeletal muscle^28^. A total of 16 samples (4 replicates per treatment group) was processed. For each sample, cyanine-3 (Cy3)-labeled cRNA was prepared from 200 ng of total RNA using the One-Color Quick Amp Labeling kit (Agilent) according to the manufacturer’s instructions, followed by the Agencourt RNAClean XP (Agencourt Bioscience Corporation, Beverly, MA, USA). Approximately 600 ng of Cy3-labeled cRNA was hybridized onto the SurePrint G3 Porcine GE microarray (8 × 60 K) following the manufacturer’s instructions. Slides were scanned immediately after washing on an Agilent G2505C Microarray Scanner using the Agilent Scan Control A.8.5.1 software, and the fluorescence signals were extracted using the Agilent Feature Extraction software v10.10.1.1 with the default parameters. The microarray data were analyzed using R (www.r-project.org, R v. 3.1.2) and the Bioconductor packages (www.bioconductor.org, v 3.0) as described in GEO entry GSE97821, which also contains all experimental details. A total of 16 samples (4 replicates per treatment group) was processed.

Network analysis and functional analysis of the DE genes were performed using the Ingenuity Pathway Analysis tool (IPA, http://www.ingenuity.com) to identify gene networks and signaling pathways affected by the mycotoxins. The IPA output included statistical assessment of the significance of gene networks and signaling pathways based on Fisher’s exact test, and only networks and pathways that presented a P value < 0.05 or a −log P value exceeding 1.30 (FDR q-values < 0.05) and a Z-score with an absolute value ≥ 2 were preserved.

### Quantitative Real-Time Polymerase Chain Reaction (qRT-PCR) analysis

The reverse transcription and real-time qPCR steps were performed using total RNA samples (n = 6 per treatment group) as previously described^63, 64^. Non-reverse transcribed RNA was used as the non-template control for verification of the genomic DNA amplification signal. The specificity of the qPCR products was assessed at the end of the reactions by analyzing the dissociation curves. Primers were purchased from Invitrogen (Invitrogen, Life Technologies Corporation, Paisley, UK). The specific sequences are provided in Table 2. The expression stability of five candidate reference genes, (Cyclophylin A, β-actin, β2-microglobulin, Ribosomal Protein L32 (RPL32), and Hypoxanthine Phosphoribosyl transferase 1 (*HPRT-1*)) was analyzed across the different experimental samples using the BestKeeper software program^65^. *RPL-32* (SD = 0.82; r = 0.971) and β2-microglobulin (SD = 0.94; r = 0.962) were identified as the best reference genes for our dataset and used to normalize the values.

**Table 2:** Primer sequences.

Gene Symbol	Gene name	Primer sequence	Accession number and reference
*NF-κB*	Nuclear Factor Kappa B Subunit 1	F: CCTCCACAAGGCAGCAAATAG	ENSSSCT00000033438 (This study)
		R: TCCACACCGCTGTCACAGA	
*TNF-α*	Tumor necrosis factor -alpha	F: ACTGCACTTCGAGGTTATCGG	NM_214022^20^
		R: GGCGACGGGCTTATCTGA	
*TNFAIP*	Tumor necrosis factor, alpha-induced protein 3	F: CAACTGGTGTCGAGAAGTGAGG	ENSSSCT00000004592 (This study)
		R: TCCCCATTCGTTTTCAGTGC	
*CXCL-2*	Chemokine (C-X-C motif) ligand 2	F: ACCAAACGGAAGTCATAGCCA	NM_001001861 (This study)
		R: TGAGACAAACTTCCTGACCATTCTT	
*CSF-2*	Interleukin 1 - alpha	F: TCAGCCGCCCATCCAA	NM_214118 (This study)
		R: AGCCCCGGTGCCATGT	
*IL-1ß*	Interleukin 1 - beta	F: GAGCTGAAGGCTCTCCACCTC	NM_001005149^61^
		R: ATCGCTGTCATCTCCTTGCAC	
*IL-8*	Interleukin 8	F: GCTCTCTGTGAGGCTGCAGTTC	NM_213867^61^
		R: AAGGTGTGGAATGCGTATTTATGC	
*IL-17A*	Interleukin 17 - alpha	F: CCAGACGGCCCTCAGATTAC	NM_001005729.1^61^
		R: GGTCCTCGTTGCGTTGGA	
*IL-22*	Interleukin 22	F: AAGCAGGTCCTGAACTTCAC	AY937228^61^
		R: CACCCTTAATACGGCATTGG	
*RPL32*	Ribosomal Protein L32	F: AGTTCATCCGGCACCAGTCA	NM_001001636^63^
		R: GAACCTTCTCCGCACCCTGT	
*β2-Microglob*	β2-Microglobulin	F: TTCTACCTTCTGGTCCACACTGA	NM_213978^63^
		R: TCATCCAACCCAGATGCA	

### ELISA and Western blot assays

Concentrations of TNF-α and IL-8 were measured in cell culture supernatants by enzyme linked immuno-absorbent assays (ELISA). Specific kits for porcine TNF-α and IL-8 (R&D Systems, Minneapolis, MN, USA) were used according to the manufactured instructions as already described^20, 66^.

The expression of phosphorylated p38 MAPK in IPEC J2 cell lysates was analyzed by western blot as already described^26^. Briefly, total proteins were separated on SDS-PAGE, transferred onto nitrocellulose membranes, incubated with Rabbit anti-phospho-p38 (Cell Signaling Technology, Danvers, MA) overnight and further incubated with CFTM770 goat anti-rabbit IgG (Biotium, Hayward, CA). Mouse anti-β-actin (Cell Signaling Technology) was used as control. Membranes were analyzed using an Odyssey Infrared Imaging System (LI-COR; ScienceTec, Les Ulis, France). The expression of phosphorylated p38 MAPK was estimated after normalization with β-actin.

### Benchmark dose analysis of PODs for the pro-inflammatory effects of FX

A benchmark dose (BMD) analysis of the point of departure (POD) for several cytokines was conducted to establish the intestinal pro-inflammatory potency of FX. The BMD is defined as the dose corresponding to a predetermined change in an adverse response (the benchmark response (BMR) compared with the response in the control group^61^. As suggested by the U.S. EPA, one standard deviation above the control mean was used to set the BMR^61^. The PROAST (Possible Risk Obtained from Animal Studies) software was used to calculate the BMDs and their lower confidence bounds (BMDLs). This procedure is extensively described elsewhere^61^. The program has been developed by the Dutch National Institute for Public Health and the Environment (RIVM) in Bilthoven and is available from the RIVM website (http://www.rivm.nl/proast).

### Statistical analysis

For the histo-pathological analysis, the values of the scores are presented as the mean ± SD of independent experiments. The scores were analyzed using normality (Shapiro-Wilk) and homogeneity (Bartlett) tests. When these two assumptions were met, the analysis of variance (ANOVA) was applied, followed by Tukey’s test; p < 0.05 was considered significant.

The microarray data were analyzed using the R Bioconductor packages and the limma lmFit function as previously described^24^. Probes with adjusted P values ≤ 0.05 (FDR correction using the Benjamini Hodgeberg procedure) were considered differentially expressed between the treated and control conditions. Hierarchical clustering was applied to the samples and the probes using the 1-Pearson correlation coefficient as the distance and Ward’s criterion for agglomeration and illustrated as a heatmap presenting gene expression profiles of selected regulated genes.

For gene expression quantification by qRT-PCR and the statistical analysis, the mRNA expression of the target genes was normalized to the expressed housekeeping genes using the REST^©^ 2009 software (Qiagen, Valencia, CA, USA), which uses the pair-wise fixed reallocation randomization test as the statistical model^61^.

## Electronic supplementary material

Table S1: Toxicological effects reported upon exposure to FX
