# Supplementary material for: Intestinal toxicity of the type B trichothecene mycotoxin fusarenon-X: whole transcriptome profiling reveals new signaling pathways
Source: Sci Rep. 2017 Aug 8;7:7530. doi: 10.1038/s41598-017-07155-2 (PMC5548841; doi:10.1038/s41598-017-07155-2)
Supplement: Supplementary file 1 — Table S1: Toxicological effects reported upon exposure to FX [file 41598_2017_7155_MOESM1_ESM.pdf]

Intestinal toxicity of the type B trichothecene mycotoxin fusarenon-X: whole transcriptome profiling reveals new signaling pathways.

Imourana ALASSANE-KPEMBI[a,b], Juliana RUBIRA GEREZ[a,c], Anne-Marie COSSALTER[a], Manon NEVES[a], Joëlle LAFFITTE[a], Claire NAYLIES[a], Yannick LIPPI[a], Martine KOLF-CLAUW[a,d], Ana Paula L. BRACARENSE[c], Philippe PINTON[a] and Isabelle P. OSWALD[a*]

[a]Toxalim, Research Center in Food Toxicology, Université de Toulouse, INRA, ENVT, INP-PURPAN, UPS, F-31027, Toulouse France

[b]Hôpital d'Instruction des Armées, Camp Guézo 01BP517 Cotonou, Bénin

[c]Laboratory of Animal Pathology, Department of Veterinary Preventive Medicine, Universidade Estadual de Londrina, Londrina, Paraná, Brazil

[d]Present address: Université de Toulouse, Ecole Nationale Vétérinaire (ENVT)

*Corresponding author: Isabelle P. OSWALD, INRA, UMR 1331 Toxalim, Research Center in Food Toxicology, 180 Chemin de Tournefeuille, BP93173, 31027 Toulouse Cedex 03, FRANCE. Isabelle.Oswald@inra.fr, Tel: 33 (0) 5 82 06 63 66

Table S1: Toxicological effects reported upon exposure to FX

Reported effect	Biological model	Exposure route and dose/concentration range	Reference
Reproductive/developmental toxicity (Apoptosis in developing brain)	Pregnant female ICR mice	3.5 mg/kg, P.O	[1]
Pro-inflammatory cytokine and chemokine regulation	10- to 12-week-old B6C3F1mice	2.5 mg/kg bw, P.O	[2]
Emesis	Mink	0.1-0.25 mg/kg bw, P.O and I.P	[3]
Anorexia	10- to 12-week-old B6C3F1mice	0.025-2.5 mg/kg, P.O and I.P	[4]
Induction of apoptosis and cytokine production	Jurkat human T-cells	62.5-500 ng/ml, in vitro	[5]
Apoptosis	Human promyelocytic leukemia cell line HL-60	0.5µg/mL, in vitro	[6]
Inhibition of T and B cell proliferation	Human peripheral blood mononuclear cells	10-5000 ng/ml in vitro	[7]
Apoptosis	In vivo :4- to 8-week-old DDD mice; and in vitro: mice thymocytes	1.5-6 mg/kg bw, I.P; 0.1 µM in vitro	[8]
Abdominal inflammation	ddY-strain mice	1-4 mg/kg bw, I.P	[9]
Decreased serum protein level	Wistar rats	1 mg/kg bw, I.P	[10]
Immunosuppression (inhibition of human lymphocyte blastogenesis)	Human lymphocytes	0.05-500 ng/ml, in vitro	[11]
Increased vascular permeability	Rabbits	10µg, SC	[12]
Immunosuppression (mitogen response, antibody response)	6-week-old BALB/c mice	25-50µg, I.P	[13]
Diarrhea	Wistar rats	1 mg/kg bw, I.P	[14]
Reproductive/developmental toxicity (abortion, inhibition of embryonal implantation)	DDD pregnant mice	0.63-2.6 mg/kg bw, subcutaneous injection (abortion); 5-20 ppm in feed	[15]
Emesis	Dogs, minks	0.3 g/kg bw, I.V (dog), 23µg/Kg P.O (mink)	[16,17]
Hypothermia	Mice	0.7-15 mg/kg, I.P	[17]
Edema	Rats	10-100 µg, Sub-plantar injection	[17]
Hypoglycemia	Mice	I.P administration of a sub-lethal dose	[18]

References

1 Sutjarit, S. *et al.* Apoptosis and gene expression in the developing mouse brain of fusarenon-X-treated pregnant mice. *Toxicol Lett* **229**, 292-302 (2014).

2 Wu, W. D. *et al.* Effects of oral exposure to naturally-occurring and synthetic deoxynivalenol congeners on proinflammatory cytokine and chemokine mRNA expression in the mouse. *Toxicol Appl Pharm* **278**, 107-115 (2014).

3 Wu, W. D. *et al.* Comparison of Emetic Potencies of the 8-Ketotrichothecenes Deoxynivalenol, 15-Acetyldeoxynivalenol, 3-Acetyldeoxynivalenol, Fusarenon X, and Nivalenol. *Toxicol Sci* **131**, 279-291 (2013).

4 Wu, W. D. *et al.* Comparison of murine anorectic responses to the 8-ketotrichothecenes 3-acetyldeoxynivalenol, 15-acetyldeoxynivalenol, fusarenon X and nivalenol. *Food Chem Toxicol* **50**, 2056-2061 (2012).

5 Pestka, J. J., Uzarski, R. L. & Islam, Z. Induction of apoptosis and cytokine production in the Jurkat human T cells by deoxynivalenol: role of mitogen-activated protein kinases and comparison to other 8-ketotrichothecenes. *Toxicology* **206**, 207-219 (2005).

6 Miura, K., Aminova, L. & Murayama, Y. Fusarenon-X induced apoptosis in HL-60 cells depends on caspase activation and cytochrome c release. *Toxicology* **172**, 103-112, (2002).

7 Berek, L., Petri, I. B., Mesterhazy, A., Teren, J. & Molnar, J. Effects of mycotoxins on human immune functions in vitro. *Toxicology in Vitro* **15**, 25-30 (2001).

8 Miura, K. *et al.* Induction of apoptosis with fusarenon-X in mouse thymocytes. *Toxicology* **127**, 195-206 (1998).

9 Matsuoka, Y. & Kubota, K. Characteristics of Inflammation Induced by Fusarenon-X, a Trichothecene Mycotoxin from Fusarium Species. *Toxicol Appl Pharm* **91**, 333-340 (1987).

10 Matsuoka, Y. & Kubota, K. Studies on Mechanisms of Diarrhea Induced by Fusarenon-X, a Trichothecene Mycotoxin from Fusarium Species - Fusarenon-X-Induced Diarrhea Is Not Mediated by Cyclic-Nucleotides. *Toxicol Appl Pharm* **91**, 326-332 (1987).

11 Forsell, J. H. & Pestka, J. J. Relation of 8-Ketotrichothecene and Zearalenone Analog Structure to Inhibition of Mitogen-Induced Human-Lymphocyte Blastogenesis. *Appl Environ Microb* **50**, 1304-1307 (1985).

12 Ueno, Y. Toxicological features of T-2 toxin and related trichothecenes. *Fundam Appl Toxicol* **4**, S124-132 (1984).

13 Masuda, E., Takemoto, T., Tatsuno, T. & Obara, T. Induction of Suppressor Macrophages in Mice by Fusarenon-X. *Immunology* **47**, 701-708 (1982).

14 Matsuoka, Y. & Kubota, K. Studies on Mechanisms of Diarrhea Induced by Fusarenon-X, a Trichothecene Myco-Toxin from Fusarium Species. *Toxicol Appl Pharm* **57**, 293-301 (1981).

15 Ito, Y., Ohtsubo, K. & Saito, M. Effects of Fusarenon-X, a Trichothecene Produced by Fusarium-Nivale, on Pregnant Mice and Their Fetuses. *Jpn J Exp Med* **50**, 167-172 (1980).

16 Male, D. *et al.* Modeling the emetic potencies of food-borne trichothecenes by benchmark dose methodology. *Food Chem Toxicol* **94**, 178-185 (2016).

17 Matsuoka, Y., Kubota, K. & Ueno, Y. General Pharmacological Studies of Fusarenon-X, a Trichothecene Mycotoxin from Fusarium Species. *Toxicol Appl Pharm* **50**, 87-94 (1979).

18 Shimizu, T., Nakano, N., Matsui, T. & Aibara, K. Hypoglycemia in Mice Administered with Fusarenon-X. *Jpn J Med Sci Biol* **32**, 189-198 (1979).
